# Hypertonic lactate for the treatment of intracranial hypertension in patients with acute brain injury

**DOI:** 10.1038/s41598-022-07129-z

**Published:** 2022-02-22

**Authors:** Adriano Bernini, John-Paul Miroz, Samia Abed-Maillard, Eva Favre, Carolina Iaquaniello, Nawfel Ben-Hamouda, Mauro Oddo

**Affiliations:** 1 Neuroscience Critical Care Research Group, Department of Intensive Care Medicine, CHUV-Lausanne University Hospital and Faculty of Biology and Medicine, 1011 Lausanne, Switzerland; 2 School of Medicine and Surgery, University of Milano-Bicocca, Milan, Italy; 3 Medical Directorate for Research, Education and Innovation, CHUV, Lausanne, Switzerland

**Keywords:** Neuroscience, Medical research

## Abstract

Hypertonic lactate (HL) is emerging as alternative treatment of intracranial hypertension following acute brain injury (ABI), but comparative studies are limited. Here, we examined the effectiveness of HL on main cerebral and systemic physiologic variables, and further compared it to that of standard hypertonic saline (HS). Retrospective cohort analysis of ABI subjects who received sequential osmotherapy with 7.5% HS followed by HL—given at equi-osmolar (2400 mOsmol/L) and isovolumic (1.5 mL/kg) bolus doses—to reduce sustained elevations of ICP (> 20 mmHg). The effect of HL on brain (intracranial pressure [ICP], brain tissue PO_2_ [PbtO_2_], cerebral microdialysis [CMD] glucose and lactate/pyruvate ratio [LPR]) and blood (chloride, pH) variables was examined at different time-points (30, 60, 90, 120 min vs. baseline), and compared to that of HS. A total of 34 treatments among 17 consecutive subjects (13 traumatic brain injury [TBI], 4 non-TBI) were studied. Both agents significantly reduced ICP (*p* < 0.001, at all time-points tested): when comparing treatment effectiveness, absolute ICP decrease in mmHg and the duration of treatment effect (median time with ICP < 20 mmHg following osmotherapy 183 [108–257] vs. 150 [111–419] min) did not differ significantly between HL and HS (all *p* > 0.2). None of the treatment had statistically significant effects on PbtO_2_ and CMD biomarkers. Treatment with HL did not cause hyperchloremia and resulted in a more favourable systemic chloride balance than HS (Δ blood chloride − 1 ± 2.5 vs. + 4 ± 3 mmol/L; *p* < 0.001). This is the first clinical study showing that HL has comparative effectiveness than HS for the treatment of intracranial hypertension, while at the same time avoiding hyperchloremic acidosis. Both agents had no significant effect on cerebral oxygenation and metabolism.

## Introduction

Contrary to chloride, lactate is a physiologic substrate^1,2^ that can be actively metabolized by the injured brain when given systemically as a supplemental hypertonic solution^3–5^. Following acute brain injury (ABI), clinical studies have shown that hypertonic lactate (HL) has positive effects on cerebral perfusion^5^, could be utilised as preferential energy substrate^3^ and might be more effective in reducing ICP than mannitol^6^. Hypertonic saline (HS) is widely used for the treatment of elevated intracranial pressure (ICP)^7–9^, with demonstrated effectiveness in reducing ICP^10^ and additional favourable effects on cerebral physiology^11–14^. However, HS has potentially detrimental systemic side-effects, mainly hyperchloremic acidosis^15^, which limits repeated administration, and warrants the identification of alternative treatments, such as HL^16^.

The objective of this study in ABI patients who received sequential osmotherapy with HS followed by HL, was to examine the effect of HL on ICP, cerebral oxygenation and energy metabolism, as well as on arterial blood pH and chloride, and to compare it to that of HS.

## Results

### Patients

Between November 2016 and December 2019, 32 consecutive ABI patients who underwent multimodal brain monitoring (including intra-parenchymal ICP, brain tissue oxygen tension [PbtO_2_] and cerebral microdialysis [CMD]) and had sustained intracranial hypertension requiring repeated osmotherapy were admitted to our department. Of these, 17 patients received sequential osmotherapy with HS as first-line treatment followed by HL as second-line agent, and were included in the present analysis. Patient baseline characteristics and outcomes are shown in Table 1. The majority of patients had TBI (13/17), all were comatose (Glasgow Coma Scale < 9) on hospital admission and had abnormal CT scan (contusions or intracranial haemorrhage). A total of 34 treatments (17 HS vs. 17 HL) were examined, administered at a median 2 days [interquartile range 1–4] from admission.

**Table 1:** Patient characteristics and outcomes.

Variable	Value
Patient number, n	17
Age, years	35 ± 16
Female gender, n (%)	5 (29)
**Diagnosis**	
Traumatic brain injury, n	13
Aneurysmal subarachnoid haemorrhage, n	3
Haemorrhagic stroke, n	1
Glasgow Coma Score on site	4 [3–7]
**Six-months Glasgow Outcome Score**^a^	
1—death, n	5
2—minimally conscious, n	0
3—severe disability, n	4
4—moderate disability, n	5
5—full recovery, n	2

^a^One patient was lost to six-month follow-up. Data are presented as mean ± standard deviation or median and interquartile range [25th–75th percentiles], unless otherwise stated.

### Effect on cerebral circulation (intracranial pressure and cerebral perfusion pressure)

**Figure 1:**
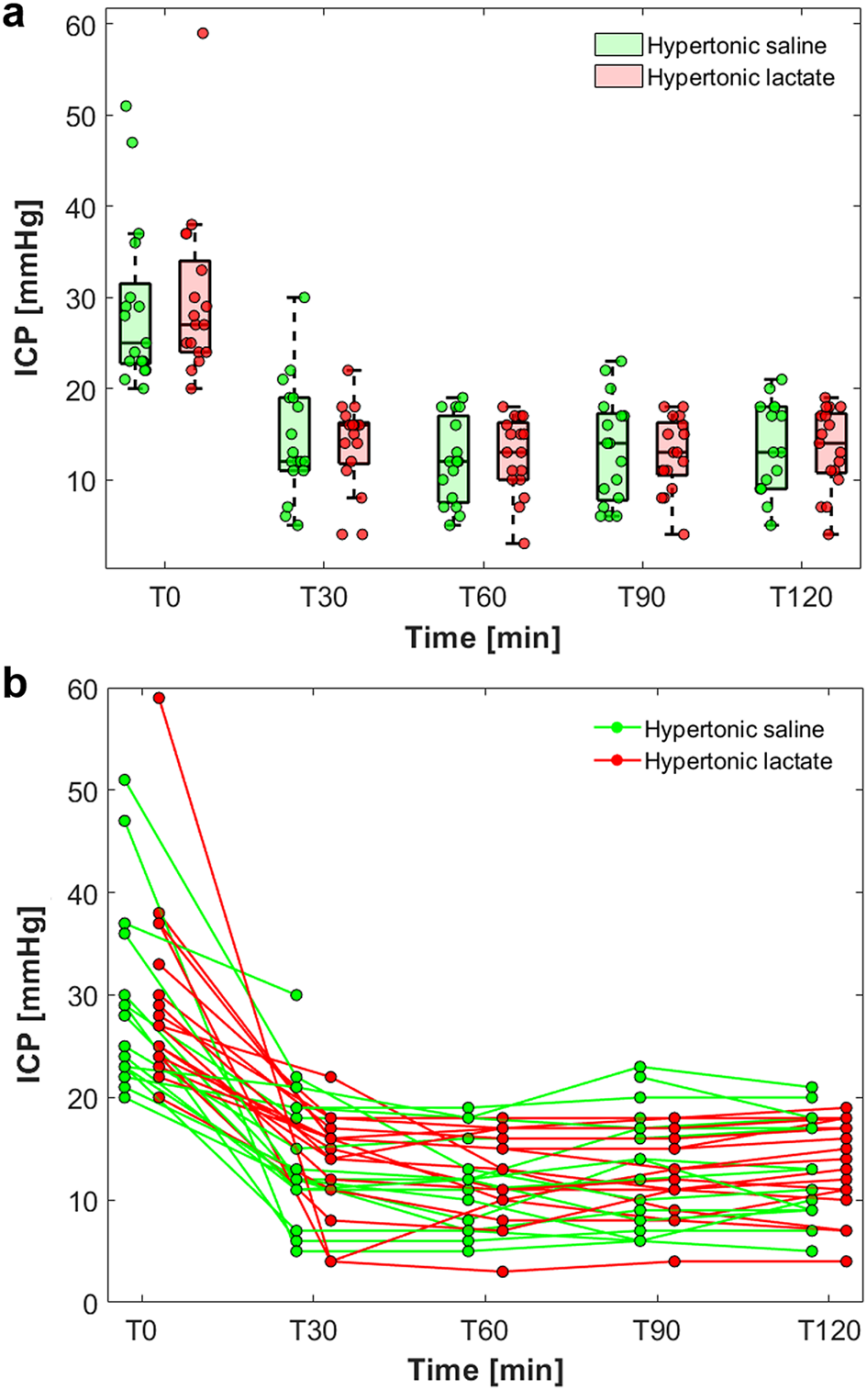
Effect of hypertonic saline and hypertonic lactate boluses on intracranial pressure (ICP) decrease. (**a**) Box-plots and scatter-plots illustrating ICP decrease over time from baseline vs. 30 (T30), 60 (T60), 90 (T90) and 120 (T120) minutes following hypertonic saline (in green) and hypertonic lactate (in red). (**b**) Spaghetti plot showing the individual patient trajectories for hypertonic saline (in green) and hypertonic lactate (in red).

Evolution of ICP over time (first 120 min) following osmotherapy with HS and HL is shown in Fig. 1. Each treatment decreased significantly ICP compared to baseline values (all *p* < 0.001; ANOVA for repeated measures). Comparisons between HL and HS did not show any statistically significant difference with respect to the absolute ICP decrease (ΔICP from baseline, in mmHg; Table 2). The duration of treatment effect was also comparable (median time spent with an ICP < 20 mmHg following osmotherapy: 183 [108–257] min with HL vs. 150 [111–419] min with HS, *p* = 0.63), as well as the comparative effect on cerebral perfusion pressure (CPP) (Table 2).

**Table 2:** Effectiveness of osmotherapy with hypertonic saline vs. hypertonic lactate on brain physiologic variables.

Variables	Hypertonic saline	Hypertonic lactate	P value
Baseline ICP (T0), mmHg	25 [23 to 33]	27 [24 to 35]	
**Post-osmotherapy**			
Δ ICP, mmHg			
T30	− 11 [− 17 to − 7]	− 13 [− 18 to − 8]	0.92
T60	− 14 [− 18 to − 9]	− 14 [− 19 to − 11]	0.92
T90	− 13 [− 19 to − 6]	− 14 [− 21 to − 11]	0.90
T120	− 12 [− 21 to − 7]	− 13 [− 21 to − 10]	0.87
Baseline MAP (T0), mmHg	93 [87 to 104]	97 [88 to 106]	
**Post-osmotherapy**			
Δ MAP, mmHg			
T30	− 9 [− 18 to − 2]	− 6 [− 15 to − 3]	0.87
T60	− 5 [− 16 to − 2]	− 10 [− 14 to − 2]	0.21
T90	− 11 [− 18 to − 5]	− 8 [− 22 to − 2]	0.63
T120	− 12 [− 16 to − 5]	− 6 [− 14 to − 2]	0.70
Baseline CPP (T0), mmHg	67 [60 to 73]	69 [62 to 78]	
**Post-osmotherapy**			
Δ CPP, mmHg			
T30	6 [− 5 to 10]	3 [0 to 11]	0.93
T60	7 [0 to 11]	4 [− 3 to 12]	0.21
T90	4 [− 7 to 8]	2 [− 6 to 15]	0.75
T120	3 [− 7 to 11]	5 [− 6 to 11]	0.91

Data are presented as median and interquartile range [25th–75th percentiles]. *P* values for comparisons of osmotherapy with hypertonic saline vs. hypertonic lactate (ANOVA for repeated measures adjusted for patient, time, treatment and time-treatment interaction).

*CPP* cerebral perfusion pressure, *ICP* intracranial pressure, *MAP* mean arterial pressure.

### Effect on brain oxygenation and energy metabolism

Complete PbtO_2_ and CMD dataset during osmotherapy was available for 11 patients (Table 3). Overall, both agents did not exert any statistically significant (and clinically relevant) effect on PbtO_2_, CMD glucose, lactate, pyruvate and lactate/pyruvate ratio. A trend towards an increase in PbtO_2_ was observed with HS in the first hour following treatment (baseline vs. 30 min, *p* = 0.09; baseline vs. 60 min, *p* = 0.06; which was not confirmed thereafter [baseline vs. 90 min and 120 min, both *p* > 0.2]; ANOVA for repeated measures). No significant changes in PbtO_2_ were observed with HL (baseline vs. each time point; all *p* > 0.2 respectively). No significant change in CMD concentrations of glucose, lactate, pyruvate and lactate/pyruvate ratio was observed with HL (baseline CMD vs. each time point; all *p* > 0.2 for all CMD variables) or HS (baseline CMD vs. each time point; all *p* > 0.2 respectively for all CMD variables, repeated measures ANOVA to compare the effect of osmotic agent on each CMD variable).

**Table 3:** Effectiveness of osmotherapy with hypertonic saline vs. hypertonic lactate on brain tissue PO_2_ and cerebral energy metabolism.

Variables	Hypertonic saline	Hypertonic lactate
**Brain tissue PO**_**2**_**, mmHg**		
Baseline	22 [14–32]	21 [18–29]
T30	24 [20–33]	22 [17–28]
T60	25 [20–33]	21 [16–26]
T90	24 [17–35]	23 [16–25]
T120	26 [18–35]	23 [17–26]
**Brain glucose, mmol/L**		
Baseline	1.3 [0.8–2.5]	0.8 [0.6–1.3]
T60	1.5 [1.1–2.4]	0.9 [0.8–1.4]
T120	1.6 [1.0–3.1]	1.0 [0.5–1.5]
**Brain lactate, mmol/L**		
Baseline	4 [2.2–7.5]	3.2 [2.6–5.8]
T60	3.3 [1.9–7.2]	3.9 [3.1–5.8]
T120	3.8 [2.1–7.7]	4 [2.8–7.2]
**Brain pyruvate, µmol/L**		
Baseline	118.1 [82.8–186.3]	128.2 [104.4–200]
T60	115.9 [77.7–149.9]	158.6 [92.8–206.2]
T120	117.7 [81–191.2]	147.5 [133.5–214.8]
**Brain lactate/pyruvate ratio**		
Baseline	33 [25–45]	27 [25–42]
T60	32 [24–43]	27 [25–35]
T120	32 [23–43]	31 [25–38]

Data are presented as median and interquartile range [25th–75th percentiles].

### Effect on arterial blood chloride and pH

Arterial blood chloride concentration did not change significantly following HL therapy (*p* = 0.21), while it increased significantly following HS (*p* < 0.001, Table 4). This resulted in a net blood chloride difference following osmotherapy of 4 ± 3 mmol/L for HS vs. − 1 ± 2.5 mmol/L for HL (*p* < 0.001).

**Table 4:** Effectiveness of osmotherapy with hypertonic saline vs. hypertonic lactate on arterial blood variables.

Variables	Baseline	Post-osmotherapy	P value
**Chloride, mmol/L**			
Hypertonic saline	111 ± 6	115 ± 6	< 0.001
Hypertonic lactate	114 ± 6	113 ± 6	0.233
**Sodium, mmol/L**			
Hypertonic saline	140.4 ± 4.2	143.4 ± 3.9	0.002
Hypertonic lactate	142.4 ± 5.1	144.7 ± 4.4	0.015
**pH**			
Hypertonic saline	7.39 ± 0.04	7.38 ± 0.05	0.269
Hypertonic lactate	7.42 ± 0.04	7.46 ± 0.03	0.009
**Lactate, mmol/L**			
Hypertonic saline	1.5 ± 1.3	1.4 ± 1.1	0.717
Hypertonic lactate	1.0 ± 0.6	2.9 ± 2.6	< 0.001
**PaCO**_**2**_**, mmHg**			
Hypertonic saline	36.4 ± 3.6	37.3 ± 3.1	0.428
Hypertonic lactate	34 ± 3.5	34.7 ± 3.3	0.537

Data are presented as mean ± standard deviation. *P* values for comparisons of baseline vs. post osmotic agent treatment (ANOVA for repeated measures).

*PaCO*_*2*_ partial pressure of carbon dioxide.

In addition, HL was associated with a significant increase in blood pH (*p* = 0.004) and lactate (*p* < 0.001), which was not the case for HS (blood pH: *p* = 0.28; and blood lactate: *p* = 0.75, Table 4). Blood sodium increased comparably with both agents (for HL: *p* = 0.01; for HS: *p* = 0.005, Table 4; net blood sodium difference: 2.7 ± 2.8 mmol/L for HL vs. 3.0 ± 1.9 mmol/L for HS, *p* = 0.72). PaCO_2_ remained stable over the treatment.

## Discussion

To date, this is the first clinical study comparing the effect of equi-osmolar and isovolumic bolus doses of HS vs. HL on cerebral and systemic physiology. The main finding of our study is that HL, when given as second-line agent to treat sustained elevated ICP, is equally effective than HS in reducing ICP. Only one previous comparative study using similar osmolar load as in our study (1283 mOsmol/L), is available in the literature, in which HL was shown to be more effective than mannitol in decreasing ICP^6^. As for HS, a meta-analysis of randomized clinical studies (total patient number 112, corresponding to 184 episodes of elevated ICP) comparing HS to mannitol found that HS was more effective than mannitol in reducing elevated ICP^10^. While of statistical significance, the net effect on ICP decrease was clinically modest (average ICP decrease favouring HS over mannitol: 2 mmHg). Here, using data from 17 patients and 34 episodes of raised ICP, we found that HL was equally effective than HS in reducing ICP, with a comparable duration of treatment effect.

Outside brain circulation and ICP/CPP, our study using a comprehensive multimodal monitoring approach provided additional information on the effect of osmotherapy with HL or HS on cerebral oxygenation and metabolism. Although no statistically significant differences were observed, our data are in line with previous findings, suggesting that HS might improve PbtO_2_^13^, while HL has an overall null effect on this variable^1,4,17^. None of the agent exerted a significant effect on CMD biomarkers of energy metabolism. Cerebral energy dysfunction, characterized by elevated CMD lactate/pyruvate ratio above normal levels (> 25) is an important pathophysiologic determinant of TBI^3,18,19^, potentially linked to mitochondrial dysfunction^20^ rather than hypoxia^21^. In our study, this seemed to be also the case, because patients had elevated lactate/pyruvate ratio, but overall PbtO_2_ was above the hypoxic threshold (15–20 mmHg). In such conditions, we previously showed that HL can be actively metabolized by the injured brain^3^ without causing “lactate flooding”^22^.

Comparison of systemic effects of HL and HS provided important findings. Looking at systemic side-effects of osmotic agents, one important limitation of HS, especially when given repeatedly, is the associated increase in blood chloride, with or without acidosis. Hyperchloremia, even at moderate levels (blood chloride ≥ 115 mmol/L) is associated with acute kidney injury and worse outcome^23,24^. Indeed, moderate hyperchloremia was observed following HS treatment in our study, which was not the case with HL. The overall net balance of blood chloride was in favour of HL, with a statistically significant difference. Recent multicentre data from general critically ill patients concluded that, compared to chloride-containing solutions, lactate-containing solutions are associated with better outcomes^25^. Our study supports the concept that HL may be a valid alternative to HS, to prevent hyperchloremic acidosis, particularly when repeated doses of osmotic agents are necessary to control elevated ICP.

Our study has some limitations. First, our data are derived from a limited patient sample size, therefore additional larger studies are required and our findings need to be considered as preliminary. However, our sample size was actually comparable to that of most previous osmotherapy studies^26–28^. Second, this was a retrospective analysis that used a within-subject design, potentially causing a selection bias and limiting the generalizability of the results. Given this limitation, for each patient the analysis was restricted only to the first treatments (i.e. first HS treatment vs. first HL treatment within the same patient), separated by at least a 2-h interval, and in conditions where no other osmotic agent (i.e. mannitol) nor any additional intervention was administered concomitantly during the 120 min treatment period. Our data could not show a difference between HS and HL in terms of effectiveness in reducing ICP but additional studies in a larger setting are needed. Third, the cohort, although consisting predominantly of TBI patients, was heterogeneous, but this was also the case for other previous reports^26,27^ and does not represent a major issue in our view. Finally, HL was used as second-line osmotic agent and was not given in parallel with HS, therefore further randomised study is needed to confirm our findings.

## Methods

### Study design and population

We retrospectively examined consecutive patients with severe ABI (November 2016–December 2019) admitted to the Department of Adult Intensive Care Medicine, Lausanne University Hospital (Centre Hospitalier Universitaire Vaudois, CHUV), Switzerland, who received both HS and HL sequentially to treat repeated episodes of elevated ICP (> 20 mmHg) and underwent cerebral multimodal monitoring with intracranial pressure (ICP) in combination with brain tissue oxygen tension (PbtO_2_) and cerebral microdialysis (CMD). Following previous studies by our group, HL has been introduced as a standard of care at our centre, to be used as second-line optional osmotherapy in alternative to HS or mannitol. Approval for retrospective data analysis with a waiver of informed consent due to the retrospective nature of the study was obtained from the local Ethical Committee of the University of Lausanne (study nr 2016-01923). The study report conforms to the STROBE statement for the report of observational cohort studies (https://www.strobe-statement.org/checklists/).

### Intracranial monitoring

ICP probe (Codman^®^, Raynham, MA, USA), PbtO_2_ probe (Licox^®^, Integra Neurosciences, Plainsboro, NJ, USA) and 20-kDa cut-off CMD catheter (CMA 70^®^, CMA Microdialysis AB, Solna, Sweden) were inserted in the operating room by a neurosurgeon and placed into the brain parenchyma (subcortical white matter). CMD samples were collected hourly and analysed immediately at the bedside using a kinetic enzymatic analyser (ISCUS Flex^®^, CMA Microdialysis AB) for extracellular concentrations of glucose, lactate and pyruvate.

### General patient management

Patients were treated according to our standard protocol. All methods were carried out in accordance with relevant guidelines and regulations^9,29,30^. All patients underwent mechanical ventilation (aiming to keep PaO_2_ and PaCO_2_ at 90–100 mmHg and 35–40 mmHg, respectively) and sedation-analgesia (with propofol infusion, at a maximal dose of 4 mg/kg/h, and sufentanil infusion, at a maximal dose of 20 µg/h). Cerebral perfusion pressure (CPP) was maintained at 60–70 mmHg, with the use of vasopressors (norepinephrine) and isotonic fluids (aiming for euvolemia). Normoglycemia (arterial blood glucose 6–8 mmol/L, with the use of continuous insulin infusion) and normothermia (core body temperature < 37.5 °C) were part of standard care.

## Osmotherapy

Treatment of intracranial hypertension (ICP > 20 mmHg for > 10 min) followed a written stepwise management algorithm, including reinforced sedation (with bolus and increasing infusion rate of propofol ± midazolam, aiming for a Richmond Agitation-Sedation Scale of − 5), moderate hyperventilation (PaCO_2_ 30–35 mmHg) and controlled normothermia (body temperature at 35–36 °C, targeted for ICP control, with the use of an automated surface cooling device). Osmotherapy was started if ICP remained > 20 mmHg despite initial management. First-line osmotic agents for the treatment of elevated ICP consisted of 7.5% HS (1283 mmol/L, or 2400 mOsmol/L; 1.5 mL/kg) or mannitol (20%, 0.5 g/kg over 20 min), given without specific recommendation on the preferred agent or order of administration, but rather based on the clinicians’ decision as *per* individualized patient needs, according to volume status and natremia. In case of repeated episodes of elevated ICP refractory to first-line osmotic agents, our management protocol allows the use of HL as second-line agent, in alternative to HS or mannitol, at the discretion of the clinician in charge of patient care. During osmotherapy, no additional pharmacological intervention was performed concomitantly. Both HS and HL were manufactured locally by the CHUV Pharmacy aiming to obtain comparable equi-osmolar (1283 mmol/L, or 2400 mOsmol/L), isovolumic (1.5 mL/kg) solutions of 7.5% HS and HL, both administered intravenously via a central venous line.

### Data extraction and processing

Patients who received both HS and HL were identified retrospectively from patient computerized medical records. Using a within-subject comparison, the main objective of this study was to examine the effectiveness of HL vs. HS on ICP decrease, defined as the absolute reduction of ICP (in mmHg) from baseline (highest ICP value within 10 min before the osmotic treatment) vs. 30, 60, 90, 120 min, and the time spent with an ICP < 20 mmHg following osmotherapy. In addition, concomitant PbtO_2_ measures were analysed. Brain metabolites (glucose, lactate, pyruvate) were sampled hourly, and analysed at baseline vs. 60 and 120 min. Blood arterial sodium and chloride, as well as other systemic variables (including pH, PaCO_2_, glucose, and lactate) pre- vs. post-osmotic treatment were also analysed for HS and HL. For each patient, only the first given bolus HS vs. first given bolus of HL was analysed. In addition, only treatments distant of at least 120 min from any other osmotic agent or concomitant pharmacologic intervention to reduce ICP (barbiturates, sedation boluses) were retained for the present analysis. The analysis was restricted to HS and HL, because treatments had equal osmolarity and volume, which was not the case for mannitol.

### Statistical analysis

Data are expressed as mean ± standard deviation or median and interquartile ranges [25th–75th percentiles], except when otherwise stated. Data distribution was tested for each variable with the Shapiro–Wilk test. Comparisons for treatment effect on brain and arterial variables were performed, for each osmotic agent, using a repeated measures ANOVA, comparing baseline (T0) vs. 30, 60, 90, 120 min. Comparisons between HL and HS were performed for absolute ICP decrease from baseline (Δ, in mmHg) at each time-point, using an ANOVA model for repeated measures, adjusting for patient, time, treatment and time-treatment interaction. Statistical analyses were conducted using JMP 14 software (JMP^®^, Cary, NC, USA). Statistical significance was set at *p* < 0.05.

## Data Availability

The datasets used and/or analysed during the current study are available from the corresponding author on reasonable request.
